# Factors Influencing Early Feeding of Foods and Drinks Containing Free Sugars—A Birth Cohort Study

**DOI:** 10.3390/ijerph14101270

**Published:** 2017-10-23

**Authors:** Diep H. Ha, Loc G. Do, Andrew John Spencer, William Murray Thomson, Rebecca K. Golley, Andrew J. Rugg-Gunn, Steven M. Levy, Jane A. Scott

**Affiliations:** 1 Australian Research Centre for Population Oral Health, School of Dentistry, The University of Adelaide, Adelaide 5005, Australia; loc.do@adelaide.edu.au (L.G.D.); spencer@adelaide.edu.au (A.J.S.); 2 Otago School of Oral Health, Dunedin 9016, New Zealand; murray.thomson@otago.ac.nz; 3 School of Pharmacy and Medical Sciences, University of South Australia, Adelaide 5005, Australia; Rebecca.Golley@unisa.edu.au; 4 School of Dental Sciences, Newcastle University, Newcastle upon Tyne NE1 7RU, UK; andrew@rugg-gunn.net; 5 College of Dentistry, University of Iowa, Iowa, IA 52242, USA; steven-levy@uiowa.edu; 6 School of Public Health, Curtin University, Perth, WA 6845, Australia; jane.scott@curtin.edu.au

**Keywords:** infant, free sugars, socioeconomic status

## Abstract

Early feeding of free sugars to young children can increase the preference for sweetness and the risk of consuming a cariogenic diet high in free sugars later in life. This study aimed to investigate early life factors influencing early introduction of foods/drinks containing free sugars. Data from an ongoing population-based birth cohort study in Australia were used. Mothers of newborn children completed questionnaires at birth and subsequently at ages 3, 6, 12, and 24 months. The outcome was reported feeding (Yes/No) at age 6–9 months of common foods/drinks sources of free sugars (hereafter referred as foods/drinks with free sugars). Household income quartiles, mother’s sugar-sweetened beverage (SSB) consumption, and other maternal factors were exposure variables. Analysis was conducted progressively from bivariate to multivariable log-binomial regression with robust standard error estimation to calculate prevalence ratios (PR) of being fed foods/drinks with free sugars at an early age (by 6–9 months). Models for both complete cases and with multiple imputations (MI) for missing data were generated. Of 1479 mother/child dyads, 21% of children had been fed foods/drinks with free sugars. There was a strong income gradient and a significant positive association with maternal SSB consumption. In the complete-case model, income Q1 and Q2 had PRs of 1.9 (1.2–3.1) and 1.8 (1.2–2.6) against Q4, respectively. The PR for mothers ingesting SSB everyday was 1.6 (1.2–2.3). The PR for children who had been breastfed to at least three months was 0.6 (0.5–0.8). Similar findings were observed in the MI model. Household income at birth and maternal behaviours were significant determinants of early feeding of foods/drinks with free sugars.

## 1. Introduction

The first years of life are a critical period in childhood development and health. Recent research has suggested that the roots of chronic conditions need to be traced back to as early in life as possible [1]. There exist disparities in health and development in the early years of life [2]. Disparities in early childhood can seem small in comparison with those among adults, but those small disparities can lead to substantially widening health trajectories later in life [3]. Targeting the causes of disparity in health and development in early life is important for the health and financial benefit for both individuals and society [4].

The oral health of children in developed countries has improved substantially over recent decades, but some groups of children in any population still carry a much larger burden of dental disease than other groups [5,6]. One reason for this uneven distribution is the role of social inequality, or socioeconomic gradients, as a fundamental determinant of health or disease [7]. The recent cross-sectional National Child Oral Health Study (NCOHS) 2012–2014 reported significant social variations in the prevalence and severity of dental caries in Australia [5]. There were strong socioeconomic status (SES) gradients in caries experience at age 5–6 years by household income (mean ratio of 3.3 in the lowest income group vs. the highest income group) and overseas-born vs. Australian-born parents (mean ratio of 1.4). It is important to understand the reasons for such SES gradients in dental caries at such an early age.

Diet is a determinant shared by oral health and weight status in childhood [8], and maternal characteristics are strong influences on child oral health [9,10] and child obesity and overweight [11,12], as well as diet, particularly in early childhood [13,14]. There is strong evidence that foods introduced in early childhood can establish preferences for tastes (e.g., sweetness) and foods consumed [15,16,17]. Through familiarisation, children develop a taste preference that in turn establishes dietary trajectories: for example, children routinely fed sugar water during infancy preferred sweeter liquids than did children who were rarely fed sugar water early in life [18,19].

The first five years of life are a time of rapid physical growth and are the years when eating behaviours develop that can serve as a foundation for future eating patterns [15]. During their early years, children are learning what, when, and how much to eat based on the transmission of cultural and familial beliefs, attitudes, and practices surrounding food and eating. In most families, women still have primary responsibility for feeding children. Mothers (and other caregivers) therefore play a vital role in determining children’s early experiences with food and eating. However, evidence also suggests that the mother’s food choice for her infant is dependent both on cultural and familial beliefs and attitudes and also on the affordability of foods [20,21].

The 2015 World Health Organization (WHO) guidelines on free sugars have identified the detrimental health effects of excess free sugars intake [22]. The WHO recommended radical measures to improve population health, with a particular focus on dental caries and obesity. It has been emphasised that the roots of the epidemics of these two conditions need to be traced back as early as possible in life so that effective preventive measures can be developed. Australian Dietary Guidelines recommend that infants under the age of 12 months are not given foods or drinks with added sugar or honey; they also recommended that fruit juice should also be avoided [23]. However, it is important to known how well the Guidelines have been adhered to, since compliance with dietary guidelines will affect some health and development outcomes [24]. The WHO Guidelines [22] and a recent systematic review [25] point to a lack of high-quality evidence of the effect of early life influences on intake of foods and drinks with free sugars . Understanding the social determinants of those early-life influences will inform measures to prevent children from developing a diet high in free sugars later in life. This study aimed to investigate the early-life socioeconomic and maternal factors that influence early feeding of foods and/or drinks with free sugars.

## 2. Methods

The present analyses used data from an ongoing population-based birth cohort study, the Study of Mothers’ and Infants’ Life Events Affecting Health (SMILE) [26]. SMILE is an observational prospective study that has recruited and is following a cohort of socioeconomically diverse South Australian newborns in Adelaide, Australia. The targeted population for the SMILE study were children born in Adelaide during the mid-2013 to mid-2014 period. All new mothers giving birth in the three major maternity hospitals in Adelaide—and who were sufficiently competent in English to be able to understand the description and instructions of the study—were invited to participate. Those mothers who indicated their intention to move out of the greater Adelaide area within a year were excluded. Mothers of newborn children were invited to participate and complete questionnaires at birth, 3, 6, 12, and 24 months. The baseline questionnaire data were collected through face-to-face interview at the time of recruitment soon after the case child’s birth. The follow-up questionnaires were administered through online, paper-based, or telephone interviews. Participating women were contacted with different means of communication in order to achieve a high level of response.

Ethics clearance was obtained from the Southern Adelaide Clinical Human Research Ethics Committee (SAC HREC 50.13) and the Women and Children Health Network Human Research Ethics Committee (HREC/13/WCHN/69), Adelaide. Mothers willing to participate had been informed about the study and provided a written consent form.

Participants who completed the six-month questionnaire (administered when children were six to nine months old) were included in this study. The primary outcome (hereafter referred as early introduction to foods/drinks with free sugars) was assessed via this questionnaire. Non-respondents were reminded via email, text message, postal letter or phone. Foods and drinks that were classified as containing free sugars (all monosaccharides and disaccharides added to foods by the manufacturer, cook or consumer, plus sugars naturally present in honey, syrups, and fruit juices) based on the WHO definition [27]. Mothers were asked the question “Has your child ever tried any of the following foods”. Mothers were asked to tick if their child had tried any of the listed 30 food/drink items. Eight items were food/drinks with added free sugars (sugar-coated cereals, biscuits/cakes, ice-cream, chocolate confectionery, chewy lollies, lollipops, cordial, and soft drinks). We also asked whether the mother/other caregiver ever put her/his child to bed with a bottle containing fruit juice and if whether she/he ever dipped a dummy in honey before giving to her child. If a parent answered affirmatively to any of these foods and drinks items (including fruit juice and honey), then the child was categorised as having early introduction to foods/drinks with free sugars. Infants who were still fed exclusively breast milk or infant formula were included in the analysis. The outcome (having early introduction to foods/drinks with free sugars) was used as a binary variable in the analysis (i.e., yes/no introduction to foods/drinks with free sugars at 6–9 months of age).

Household income was collected at the recruitment interview. Annual before-tax income was assessed by the question “Which category does your household income fall into?” The response options were categorised into approximate quartiles: Q1 (Lowest): <AU$40,000; Q2: AU$40,000 to <AU$80,000; Q3: AU$80,000 to <AU$140,000; and Q4 (Highest): AU$140,000+.

Potential early life socioeconomic factors and covariates were selected a priori. Potential factors collected at the baseline (birth) and subsequent interviews included: mother’s age at child’s birth (≤24 years; 25–34 years; 35+ years), mothers’ Indigenous status, mother’s country of birth, and mother’s education, mother’s relationship status (partner; no partner), mother’s work status, and the total number of children in the household, including the participating child. Mothers were categorised as Indigenous if they classified themselves as Aboriginal or Torres Strait Islander or both. Four categories of countries of birth were created (Australia, New Zealand, and UK; India; Asia other; or all other countries). Mothers’ educational attainment responses were used to group the mothers into three groups: school education, vocational training, and some or complete university education. Mother’s work status prior to this child’s birth was grouped into four categories: unemployed or home duties, self-employed or pensioner, part-time work, and full-time work.

Mothers were asked whether and how often they consumed soft drinks containing sugar (such as Coke, Pepsi, energy drinks). Answers were in three categories: every day, sometimes, never. Other behaviours, such as consumption of fresh fruits by the mothers and child breastfeeding were covariates. Fruit consumption was reported as every day, sometimes, or never. Breastfeeding at three months was used as a binary variable (yes or no). Exclusive breastfeeding or breastfeeding with another forms of infant feeding were categorised as “yes breastfeeding” group, and children without breastfeeding at three months were in “no breastfeeding” group.

To minimise bias, missing data on exposure variables were imputed under the assumption that data were missing at random. Twenty imputed data sets were generated using SAS Proc MI (SAS 9.4, SAS Institute Inc., Cary, NC, USA) and the results of the imputed analyses were combined following the standard methods [28,29]. In accordance with best practice, the imputation model included exposure and co-variable data, as well as the following predictors of missing values: mothers’ smoking status during pregnancy, and mothers’ obesity. The distributions of variables in the imputed data sets were consistent with the complete case data.

Generalised regression models were used to estimate association between the exposure variables (household income, mother’s SSB consumption, other maternal factors), and the outcome (early introduction to foods or drinks with free sugars) using a log-Poisson link function with robust error estimation to estimate adjusted prevalence ratio (PR) and associated 95% confidence intervals (95% CI). The models were built based on priori assumptions of associations between the explanatory variables and the outcome. The models also included the child’s age in months, calculated when the questionnaire used to assess the outcome was completed. Interactions between covariates were not included in the models. Models are presented as crude estimates and adjusted for the covariates identified above. For both the complete case model and the model with imputation, two sets of models were generated. Model 1 included all socioeconomic factors while model 2 included three additional variables: mothers’ soft drink intake, mothers’ fruit consumption, and child breastfeeding at age three months. Attenuation of estimates was evaluated. All data analyses were conducted using SAS version 9.4 (SAS Institute Inc., Cary, NC, USA).

## 3. Results

A total of 1479 children (70% of the total sample) had their six-month questionnaire returned. The age of the majority of children at the date of return of the questionnaire ranged from six to nine months. The sociodemographic characteristics of the cohort at baseline and the respondent sample are compared in Table 1. In the respondent sample at six months, there had been higher attrition of mothers from low income households, from the youngest age group, from mothers who had completed school education only, and from Indigenous mothers. The characteristics of the imputed sample are consistent with those shown in the respondent sample. Some 6% of mothers reported consuming soft drinks every day, while 46% never consumed soft drinks. Over one-third of mothers did not consume fruit daily. Over two-thirds of the infants were exclusively or partially breastfed at the age of three months.

One in five infants had had introduction to foods/drinks with added free sugars by the age of six to nine months (Table 2). In the bivariate analysis, there was a clear gradient by income in the consumption of foods/drinks with free sugars. Some 37% of mothers from the lowest income group had introduced foods or drinks with free sugars to their child, while 11% of mothers in the highest household income group did so. Those mothers who never consumed soft drinks were less likely to introduce foods or drinks with free sugars to their child than those who consumed soft drinks.

In the bivariate analysis, early introduction to foods or drinks with free sugars was also positively associated with the total number of children in the family and lack of breastfeeding at age three months. Young maternal age, low education attainment, and low fruit consumption were also positively associated with early introduction of foods or drinks with free sugars. India-born mothers were more likely to introduce their child to foods or drinks with free sugars early than mothers born in other countries. Mothers’ Indigenous status or work status prior to the birth of the child were not associated with early introduction of foods or drinks with free sugars.

Table 3 presents adjusted prevalence ratios (PR) of having early introduction to food or drinks with free sugars. Both complete case models and models with imputation showed that mothers in the two lowest income quartiles were more likely to introduce their infants to foods or drinks with free sugars than those with the highest income. The adjusted PRs for the lowest family income quartile attenuated after adjusting for maternal behaviours and child breastfeeding status but remained statistically significant. Indian born mothers, young mothers and families with three or more children were more likely to have the early introduction of foods/drinks containing free sugars.

When introduced to the models, maternal soft drink consumption, and child breastfeeding status by age three months were associated with early introduction of sugary foods/drinks (Table 3). Children of mothers who consumed soft drinks every day were 1.8 times as likely to be introduced to foods or drinks with free sugars as those of mothers who never consumed soft drinks. In contrast, children who were breastfed at age three months were less likely to be introduced to foods or drinks with free sugars by age six to nine months.

## 4. Discussion

This population-based birth cohort study provides strong evidence for SES and selected maternal factors influencing the early introduction to infants of foods or drinks that contain free sugars. Despite the Australian Infant Feeding Guidelines which advise that infants should not be introduced to foods or drinks containing added free sugars during the first year of life, one in five infants had consumed those by the age from six to nine months. The study has demonstrated important socioeconomic gradients in such practices that are not fully explained by maternal health behaviours.

Information on the prevalence of early introduction to free foods/drinks to infants is scarce and varied in nature. Cross-sectional data from the US NHANES (2009–2012) showed that some 44% of 6–11-month-old children had sweet snacks and desserts and one in seven had had sweetened beverages [30]. Another cross-sectional study, the 2008 Feeding Infants and Toddlers study, reported that 17% of US children aged 6–9 months had any type of dessert, sweet, or sweetened beverage [31].

Little is known about what Australian infants and toddlers consume, since neither the 2011–2012 Australian National Nutrition and Physical Activity Survey [32] nor the 2007 Australian National Children’s Nutritional and Physical Activity Survey [33] investigated the diets of children younger than two years of age. The limited available data come from a relatively small number of single-centre or regional studies but they support the international studies’ finding that a substantial proportion of infants and toddlers are exposed early in life to foods and beverages with ‘added sugar’. For instance, a population-based cohort study of 587 infants in Peth reported that, by five and half months of age, 21% had been exposed to biscuits and cakes, 11% to ice cream, and 5% to sugar-sweetened beverages. By six and half months of age, these percentages had increased to 57%, 34%, and 9%, respectively [34]. A more recent Australian study of 551 children 12–16 months of age reported that 21% of children had eaten sweet biscuits in the preceding 24 hours [35]. A study of 374 Australian children reported that children aged 12–36 months consumed a variety of sweet snacks and drinks; some 35% consumed sweet biscuits, cake, or muffins; lollies and soft drinks were consumed by 26% and 9% of children, respectively [36]. Our large population-based cohort study confirmed that early introduction of foods or drinks with free sugars was common in children under 12 months of age, contrary to the Australian dietary guidelines for infants [23].

The observed early introduction of foods or drinks with free sugars can lead to negative consequences later in life [37]. There is strong evidence that foods introduced in early childhood can establish preferences for tastes (e.g., sweetness) and foods [38], which have been shown to track into later life [39,40]. Some Australian evidence for tracking of dietary patterns, from a longitudinal study of 177 Melbourne children, which indicated that, by nine and 18 months, 38% and 86% of children had consumed sweet energy-dense snacks, respectively. Being a consumer of sweet energy-dense snacks at nine months was a strong independent predictor of greater intake nine months later [41]. Similarly, Park et al. (in a longitudinal US study) reported that, when compared with children who had not consumed sugar-sweetened beverage (SSB) before 12 months, those who had consumed SSB before six months were twice as likely to consume SSB at least once a day by 12 months of age after adjustment for maternal characteristics and other potential influences [19]. Early intake of free sugars has been found to predict diet and weight outcomes by the age of six years among US children [42,43].

The effects of diet on dental caries have been documented in reviews of mostly cross-sectional studies [25]. Infant feeding practices have been shown to be associated with the development of early childhood caries; in particular, prolonged bottle-feeding, and the addition of SSB to feeding bottles [44,45]. The consumption of SSB during infancy has been found to be associated with dental caries at early school age [46].

An important finding of the current study was a strong and independent effect of socioeconomic factors on the early introduction of foods or drinks with free sugars. Children born to low-income mothers were more likely to be introduced early to those. This fact is likely to lead to widening inequality in health later in the life course [2]. This study has contributed high quality evidence collected from a population-based research to understand early life influences of dietary factors.

The strength of the current study lies in the population representativeness of the study sample [26], which allows for generalisation of the findings. Anticipating relatively higher long-term attrition by participants in the low socioeconomic groups, we recruited slightly more children born to mothers from those population groups. The retention rate was relatively high, further strengthening the sample’s representativeness and statistical power. The high number of mother/child dyads this cohort makes it one of the largest studies internationally among those investigating factors influencing child oral health. Another strength is the conduct of both complete case and multiple imputation analysis. Diagnostic comparisons of the observed and imputed data confirmed credibility of the analysis [47].

Our study findings could underestimate the true prevalence of early feeding of any foods or drinks with free sugars to some extent, because a small number of foods or drinks containing free sugars were not included. We did not collect information on homemade desserts that might have free sugars. We also did not ask if free sugars were added to infant formula. While uncommon, this practice might occur in some communities. However, inclusion of such a practice would likely further widen the observed socioeconomic differences in feeding infants with foods or drinks with added free sugars. Another limitation was that the outcome was reported for the age range from six to nine months. The range reflected the lag time in gaining a response to the questionnaire across several follow-up attempts. However, this age range is still within the age of 12 months covered by the Australian Infant Feeding Guidelines [23].

## 5. Conclusions

Our finding suggest that dietary risk factors for dental caries and obesity/overweight can start early in life. Further, the observed socioeconomic inequality in this early exposure to sugar could lead to widening socioeconomic inequality in these conditions later in life. Our longitudinal cohort study will allow examination of the long-term effect of such early introduction to free sugars on childhood caries and obesity/overweight when the children grow up. Targeting low income mothers during pregnancy and at the birth of their child with appropriate dietary advice could lead to reduction in the variation in early intake of sugary foods/drinks among very young children.

## Figures and Tables

**Table 1:** Distribution of the exposures and sample characteristics.

Characteristics	Sample at Baseline (%)	Respondent Sample (*n*, %)		Imputed Sample (*n*, %)	
**Household income**		1408		1479	
Q1 (lowest) (≤AU$40,000)	19.6		15.1		15.1
Q2 (AU$40,000–80,000)	34.3		33.4		33.4
Q3 (AU$80,000–120,000)	27.6		29.1		29.1
Q4 (highest) (AU$120,000+)	18.5		22.4		22.4
**Mother’s soft drink consumption**		1207			
Everyday	5.9		5.8		5.9
Sometime	48.7		48.6		48.6
Never	45.4		45.6		45.5
**Breastfeeding at 3 months**		1418			
Yes	72.0		72.9		72.9
No	28.0		27.1		27.1
**Mother’s fruit consumption**		1206			
Everyday	65.0		64.9		64.7
Sometime/Never	35.0		35.1		35.3
**Mother’s age at child birth (years)**		1479			
≤24 years	16.3		12.5		-
25–34 years	64.2		67.1		-
35+ years	19.5		20.4		-
**Maternal education completed**		1467			
School	26.8		21.3		21.3
Vocational	27.2		26.2		26.2
Some university or higher	46.0		52.5		52.5
**Mother’s country of birth**		1462			
Australia, NZ and UK	73.0		75.0		75.0
Asia-other	11.4		11.1		11.1
India	8.9		7.8		7.8
Other	6.7		6.1		6.1
**Indigenous status**		1444			
Yes	2.5		1.2		1.2
No	97.5		98.8		98.8
**Single parent status**		1466			
Yes	8.0		6.5		6.5
No	92.0		93.5		93.5
**Total number of children**		1433			
3+ children	18.8		48.2		48.4
2 children	36.0		35.5		35.4
1 child	45.2		16.3		16.2
**Mother’s work status prior to the birth**		1456			
Unemployed/home duties	28.8		25.6		25.7
Self-employed/pensioner	5.1		4.6		4.6
Part-time	29.6		30.6		30.6
Full-time	36.5		39.2		39.1

SEIFA: Socioeconomic Index for Areas.

**Table 2:** Percentage of children who experienced early introduction of foods or drinks with free sugars, by socioeconomic status (SES) and other covariates.

Characteristics	Complete Case		With Imputation	
	Per Cent	95% CI	Per Cent	95% CI
Total % with Early Introduction of Foods or Drinks with Free Sugars	21.4	19.1‒23.7		
**Household income**				
Q1 (lowest) (≤AU$40,000)	35.9	29.4‒42.3	37.0	29.8‒44.7
Q2 (AU$40,000–80,000)	23.8	20.0‒27.7	25.5	21.3‒30.1
Q3 (AU$80,000–120,000)	18.3	14.6‒22.0	17.8	14.1‒22.1
Q4 (highest) (AU$120,000+)	11.4	8.1‒15.4	11.0	7.6‒15.2
**Mother’s soft drink consumption**				
Everyday	42.9	31.1‒55.3	43.7	31.9‒56.0
Sometime	23.6	20.1‒27.0	23.3	20.0‒26.9
Never	16.3	13.3‒19.7	16.6	13.6‒19.9
**Breastfeeding at 3 months**				
Yes	16.5	14.3‒19.0	17.3	14.9‒19.9
No	33.9	29.1‒38.8	33.9	28.6‒39.5
**Mother’s fruit consumption**				
Everyday	18.1	15.4‒20.8	18.1	15.5‒20.9
Sometime/Never	27.7	23.5‒32.2	26.9	22.6‒31.5
**Maternal age at birth (years)**				
≤24 years	34.3	27.3‒41.2	36.0	28.3‒44.2
25–34 years	20.8	18.3‒23.5	20.6	17.9‒23.6
35+ years	15.9	11.8‒20.1	15.4	11.2‒20.5
**Maternal education attainment**				
School	27.8	22.8–32.8	27.7	22.8‒32.7
Vocational	24.7	20.4‒29.1	24.6	20.3‒28.8
Some university or higher	17.5	14.9‒20.2	17.4	14.8‒20.1
**Mother’s country of birth**				
Australia, NZ and UK	21.2	18.8‒23.7	20.7	18.1‒23.5
Asia-other	17.9	12.3‒24.7	19.0	12.9‒26.5
India	35.1	26.4‒44.6	36.0	26.5‒46.8
Other	16.9	9.1‒26.3	17.8	9.8‒28.5
**Indigenous**				
Yes	35.3	14.2‒61.7	38.4	12.0‒64.9
No	21.6	19.5‒23.8	21.3	19.0‒23.6
**Single parent**				
Yes	32.6	23.4‒42.0	32.0	21.4‒42.6
No	20.8	18.7‒23.0	20.7	18.3‒23.0
**Total number of children**				
3+ children	30.0	24.2‒36.4	32.8	26.1‒39.5
2 children	20.0	16.7‒23.8	19.4	15.6‒23.1
1 child	19.7	16.8‒22.9	19.4	16.3‒22.6
**Mother’s work status prior to the birth**				
Unemployed/home duties	27.9	23.4‒32.7	27.8	22.8‒33.0
Self-employed	16.4	7.6‒25.3	17.2	7.5‒27.0
Part-time	21.5	17.8‒25.6	21.6	17.4‒25.7
Full-time	18.3	15.2‒21.7	17.9	14.5‒21.3

Per cent: Percentage of children introduced to foods or drinks during age six to nine months. 95% CI: 95% confidence intervals of per cent.

**Table 3:** Multivariable regression model for early introduction to foods or drinks with free sugars.

Characteristics	Complete Case Models				Models with Imputation			
	Model 1		Model 2		Model 1		Model 2	
	PR	95% CI	PR	95% CI	PR	95% CI	PR	95% CI
**Household Income**								
Q1 (lowest) (≤AU$40,000)	2.3	1.4–3.8	1.9	1.2–3.1	2.0	1.3–3.1	1.8	1.2–2.9
Q2 (AU$40,000–80,000)	1.8	1.2–2.8	1.8	1.2–2.6	1.6	1.1–2.4	1.6	1.1–2.4
Q3 (AU$80,000–120,000)	1.5	1.0–2.3	1.4	0.9–2.1	1.4	1.0–2.1	1.4	0.9–2.1
Q4 (highest) (AU$120,000+)	Ref		Ref		Ref		Ref	
**Mother’s country of birth**								
Other	1.0	0.6–1.7	1.3	0.7–2.3	0.9	0.5–1.6	1.0	0.6–1.7
Asia-other	1.0	0.7–1.6	1.1	0.7–1.8	1.0	0.7–1.5	1.1	0.7–1.6
India	2.1	1.4–3.3	2.1	1.3–3.6	2.1	1.4–3.1	2.2	1.5–3.4
Australia, NZ and UK	Ref		Ref		Ref		Ref	
**Total number of children**								
3+ children	1.8	1.3–2.6	1.9	1.3–2.9	1.8	1.3–2.5	1.8	1.3–2.5
2 children	1.1	0.8–1.4	1.0	0.7–1.5	1.1	0.8–1.4	1.1	0.9–1.5
1 child	Ref		Ref		Ref		Ref	
**Maternal age at birth (years)**								
≤24 years	2.1	1.3–3.3	1.9	1.1–3.2	1.9	1.3–3.0	1.7	1.1–2.6
25–34 years	1.3	0.9–1.8	1.3	0.8–1.9	1.3	0.9–1.8	1.2	0.9–1.7
35+ years	Ref		Ref		Ref		Ref	
**Mother’s soft drink consumption**								
Everyday			1.8	1.1–2.9			1.7	1.1–2.5
Sometime			1.2	0.9–1.6			1.1	0.9–1.4
Never			Ref				Ref	
**Mother’s fruit consumption**								
Every day			0.8	0.6–1.0			0.8	0.6–1.0
Never/Sometime			Ref					
**Breastfeeding at 3 months**								
Yes			0.6	0.5–0.8			0.6	0.5–0.8
No			Ref				Ref	

PR: Prevalence ratios. 95% CI: 95% confidence interval. Model 1 adjusted for the presented variables plus age in months when the questionnaire used to assess the outcome was completed, mother’s education, Indigenous status, and work status; Model 2 adjusted for variables in the model 1 and other covariates.
